# “OLDER ADULTS SHOULD…”: CROSS-NATIONAL DIFFERENCES IN ENDORSEMENT OF PRESCRIPTIVE AGE STEREOTYPES

**DOI:** 10.1093/geroni/igad104.0892

**Published:** 2023-12-21

**Authors:** M Clara P de Paula Couto, Helene Fung, Sylvie Graf, Thomas M Hess, Shyhnan Liou, Jana Nikitin, Klaus Rothermund

**Affiliations:** Friedrich Schiller University Jena, Jena, Thuringen, Germany; The Chinese University of Hong Kong, Hong Kong, Hong Kong; Czech Academy of Sciences, Brno, Moravskoslezsky kraj, Czech Republic; NC State University, Raleigh, North Carolina, United States; National Cheng Kung University, Tainan, Tainan, Taiwan (Republic of China); University of Vienna, Vienna, Wien, Austria; Friedrich Schiller University Jena, Jena, Thuringen, Germany

## Abstract

Normative expectations about how older adults should behave are known as prescriptive age stereotypes (or “prescriptive views of aging,” PVoA). Previous research has shown that endorsement of PVoA varies across age groups but has not yet examined the variability of PVoA endorsement across countries. Considering that context may influence the endorsement of PVoA, we investigated differences in endorsement reported by an international sample of adults (N = 2,902) from the Aging as Future study covering the age range from 40 to 90 years in five countries: Czechia, Germany, Hong Kong, Taiwan, and the United States (US). We focused on endorsement of two types of PVoA, that is, disengagement (older adults should make way for the younger generation), and activation (older adults should remain socially engaged). Overall, participants reported a stronger endorsement of activation compared to disengagement (i.e., a focus on activation). Replicating previous studies, compared to young and middle-aged adults, older adults more strongly endorsed PVoA. Most importantly, cross-national differences emerged, indicating that overall endorsement of PVoA (averaged across activation and disengagement) was the strongest in Taiwan and the weakest in Czechia and Germany. However, the focus on activation (vs. disengagement) was the highest in the US followed by Hong Kong and Germany, and then by Czechia and Taiwan. Cross-national differences in the belief that the State should support older adults, in cultural values, and in views of own aging predicted the focus on activation and partially explained differences in the strength of this focus between countries.

